# The complete mitochondrial genome of *Cephenemyia stimulator* (Diptera: Oestridae)

**DOI:** 10.1080/23802359.2021.1945969

**Published:** 2021-09-15

**Authors:** Gaël Aleix-Mata, Ana María López-Beceiro, Luis E. Fidalgo, Jesús M. Peréz, Antonio Sanchéz

**Affiliations:** a Department of Experimental Biology, Jaén University, Jaén, Spain; b Department of Animal and Plant Biology, and Ecology, Jaén University, Jaén, Spain; c Department of Anatomy, Animal Production and Veterinary Clinical Sciences, University of Santiago de Compostela, Lugo, Spain; d Wildlife Ecology & Health group (WE&H), Jaén, Spain

**Keywords:** Complete mitochondrial genome, *Cephenemyia stimulator*, Oestridae, Diptera

## Abstract

*Cephenemyia stimulator* parasitizes roe deer (*Capreolus capreolus*) throughout its geographical distribution. The complete circular *C. stimulator* mitogenome was assembled, which is 16,407 bp in length, and encodes 13 protein-coding genes, 22 tRNA genes, and 2 rRNA genes. A phylogenetic tree was built with mitogenome sequences, including *C. stimulator* and 13 related Oestridae species, using *Sarcophaga tuberosa* as an outgroup.

Genus *Cephenemyia* includes several species parasitizing cervid hosts. *Cephenemyia stimulator* (Hunter, 1916) can be found in the nasal cavity, pharynx, and throat of roe deer, *Capreolus capreolus*, throughout its geographical range (Zumpt 1965; Colwell et al. 2006; Scholl et al. 2019). In this study, we assembled and analyze for the first time the complete mitochondrial genome of *C. stimulator.*

Larvae of *C. stimulator* were obtained during the necropsy of a roe deer (*Capreolus capreolus*) (Lugo, Spain: 43°02′04.7′′N 7°38′31.1′′W). The specimens were deposited at the Department of Experimental Biology of the University of Jaén (https://www.ujaen.es/departamentos/bioexp; Contact email: abaca@ujaen.es) under the references 713-1 to 713-10. Larvae were identified based on morphological criteria, in particular, the posterior peritremes and dorsal and ventral spinulation (Zumpt 1965; Colwell et al. 2006). The total DNA was extracted from half larvae of the specimen 713-5 with the Quick-DNA Tissue/Insect kit (Zymo Research) and 20 Gbp of sequences were obtained using the Illumina^®^ Hiseq™ 2000 platform in paired-end reads with length 2 × 100 nt. We first performed a quality trimming with Trimmomatic (Bolger et al. 2014) to get complete read pairs with quality higher than Q20 in the whole read after removing adapters and used a million read pairs selected randomly with seqTK (https://github.com/lh3/seqtk). The mitogenome was assembled using the MITObim v1.8 program (Hahn et al. 2013) with the ‘–quick’ option with a maximum mismatch of 15%, and using as reference the mitogenome of *Cephenemyia trompe* (accession number MN814272; Li et al. 2020). The *C. stimulator* mitogenome was annotated with MITOS (Bernt et al. 2013) and tRNA scan-SE (Lowe and Eddy 1997), and annotations of the genes were refined by manual comparison with the *C. trompe* mitogenome (Li et al. 2020).

The length of the mitochondrial genome is 16,407 bp, with an overall 77.4% AT content (GenBank accession number MW145178). The nucleotide distribution for the mitochondrial genome is 39.5% A, 14.2% C, 8.4% G, and 37.9% T. It contains typically 37 genes (13 protein-coding genes, 22 transfer RNAs, 2 ribosomal RNAs) and one control region (D-loop). The mitochondrial genome of *C. trompe* is the only one available for the *Cephenemyia* genus (Li et al. 2020). In comparison, these two *Cephenemyia* mitogenomes are very similar in organizations and in length, 16,407 bp and 16,387 bp respectively for *C. stimulator* and *C. trompe*, and have a percentage of identity of 97.7%.

In addition, we analyzed the phylogenetic relationships of *C. stimulator*. The phylogenetic analysis was performed aligning the sequences with Clustal Omega (Sievers et al. 2011), the poorly aligned positions and divergent regions were removed using Gblocks program v0.91b (Talavera and Castresana 2007). The phylogenetic tree was built using the Maximum Likelihood method with 1000 replicates with MEGAX software (Kumar et al. 2018). For this analysis, the mitochondrial genome of the 13 Oestridae species currently available in GenBank were used, and the genome of *Sarcophaga tuberosa* (Zhang et al. 2019; MK820723) as outgroup. The results showed a conventional taxon pattern, previously established for the Oestridea family, and shows *C. stimulator* in the same branch that *C. trompe,* the sister group of the genera *Estrus* and *Rhinoestrus* (Figure 1).

**Figure 1.**
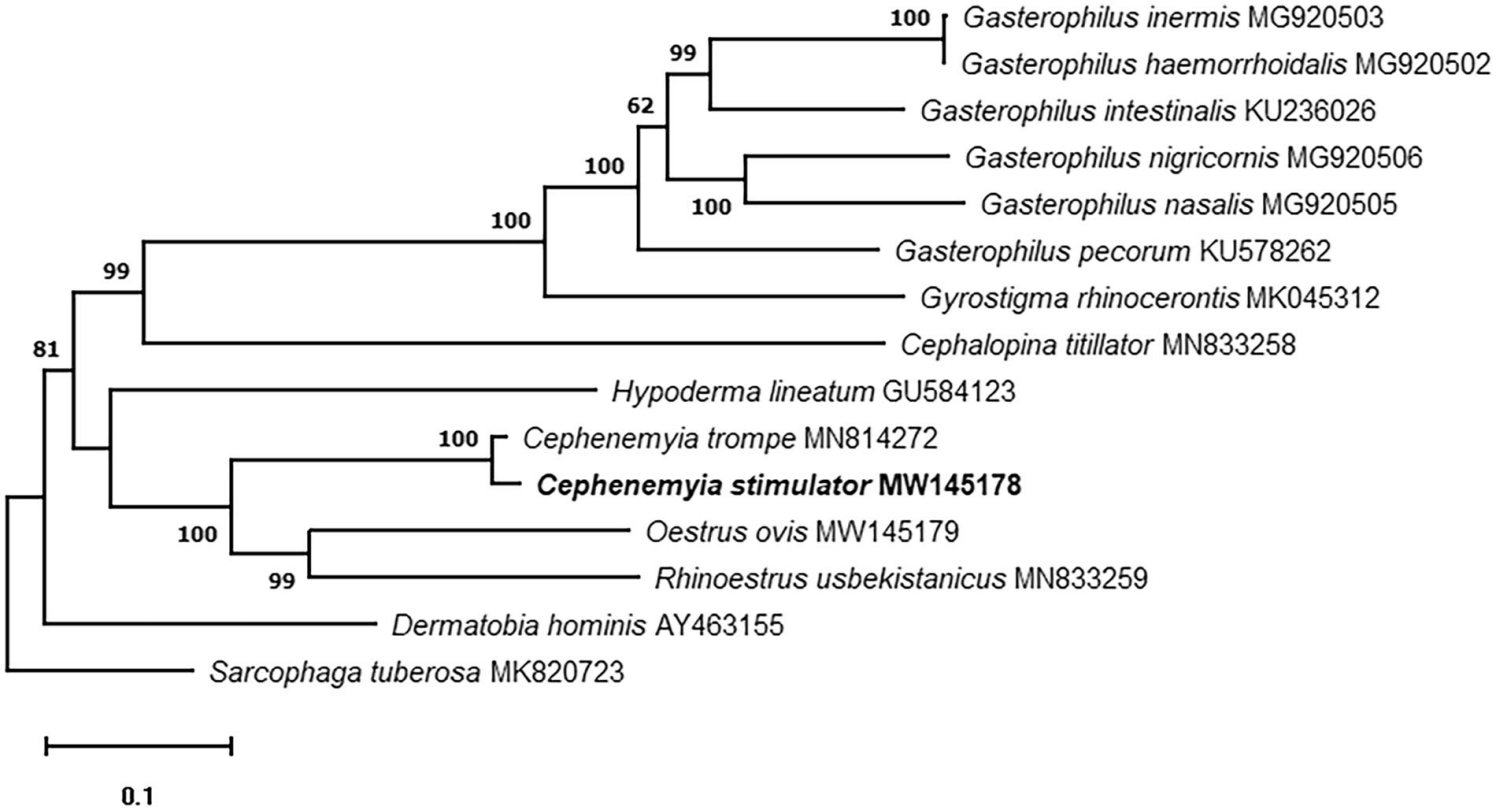
Maximum-likelihood phylogenetic tree based on the complete mitochondrial genomes of 13 Oestridae species and *Sarcophaga tuberosa* as out of group. Nodal numbers represent bootstrap support with 1000 bootstrap replicates.

## Data Availability

The data that support the findings of this study are openly available in the US National Center for Biotechnology Information (NCBI database) at https://www.ncbi.nlm.nih.gov/, reference number: MW145178. Raw sequencing reads used in this study were deposited in the public repository BioSample with accession number SAMN19601264 (https://www.ncbi.nlm.nih.gov/biosample/19601264).
